# Imaging long distance propagating calcium signals in intact plant leaves with the BRET-based GFP-aequorin reporter

**DOI:** 10.3389/fpls.2014.00043

**Published:** 2014-02-18

**Authors:** Tou Cheu Xiong, Elsa Ronzier, Frédéric Sanchez, Claire Corratgé-Faillie, Christian Mazars, Jean-Baptiste Thibaud

**Affiliations:** ^1^Biochimie et Physiologie Moléculaire des Plantes, Institut National de la Recherche Agronomique, UMR 386Montpellier, France; ^2^Biochimie et Physiologie Moléculaire des Plantes, Centre National de la Recherche Scientifique, UMR 5004Montpellier, France; ^3^Biochimie et Physiologie Moléculaire des PlantesSupAgro, Montpellier, France; ^4^Biochimie et Physiologie Moléculaire des Plantes, UM2Montpellier, France; ^5^Laboratoire de Recherche en Sciences Végétales, Université de Toulouse, UPS, UMR 5546Castanet-Tolosan, France; ^6^Centre National de la Recherche Scientifique, UMR 5546Castanet-Tolosan, France

**Keywords:** *Arabidopsis thaliana*, calcium imaging, cooled CCD camera, GFP-aequorin, leaf, long distance calcium signaling, calcium waves, salt stress

## Abstract

Calcium (Ca^2+^) is a second messenger involved in many plant signaling processes. Biotic and abiotic stimuli induce Ca^2+^ signals within plant cells, which, when decoded, enable these cells to adapt in response to environmental stresses. Multiple examples of Ca^2+^ signals from plants containing the fluorescent yellow cameleon sensor (YC) have contributed to the definition of the Ca^2+^ signature in some cell types such as root hairs, pollen tubes and guard cells. YC is, however, of limited use in highly autofluorescent plant tissues, in particular mesophyll cells. Alternatively, the bioluminescent reporter aequorin enables Ca^2+^ imaging in the whole plant, including mesophyll cells, but this requires specific devices capable of detecting the low amounts of emitted light. Another type of Ca^2+^ sensor, referred to as GFP-aequorin (G5A), has been engineered as a chimeric protein, which combines the two photoactive proteins from the jellyfish *Aequorea victoria*, the green fluorescent protein (GFP) and the bioluminescent protein aequorin. The Ca^2+^-dependent light-emitting property of G5A is based on a bioluminescence resonance energy transfer (BRET) between aequorin and GFP. G5A has been used for over 10 years for enhanced *in vivo* detection of Ca^2+^ signals in animal tissues. Here, we apply G5A in Arabidopsis and show that G5A greatly improves the imaging of Ca^2+^ dynamics in intact plants. We describe a simple method to image Ca^2+^ signals in autofluorescent leaves of plants with a cooled charge-coupled device (cooled CCD) camera. We present data demonstrating how plants expressing the G5A probe can be powerful tools for imaging of Ca^2+^ signals. It is shown that Ca^2+^ signals propagating over long distances can be visualized in intact plant leaves and are visible mainly in the veins.

## Introduction

**Figure 1:**
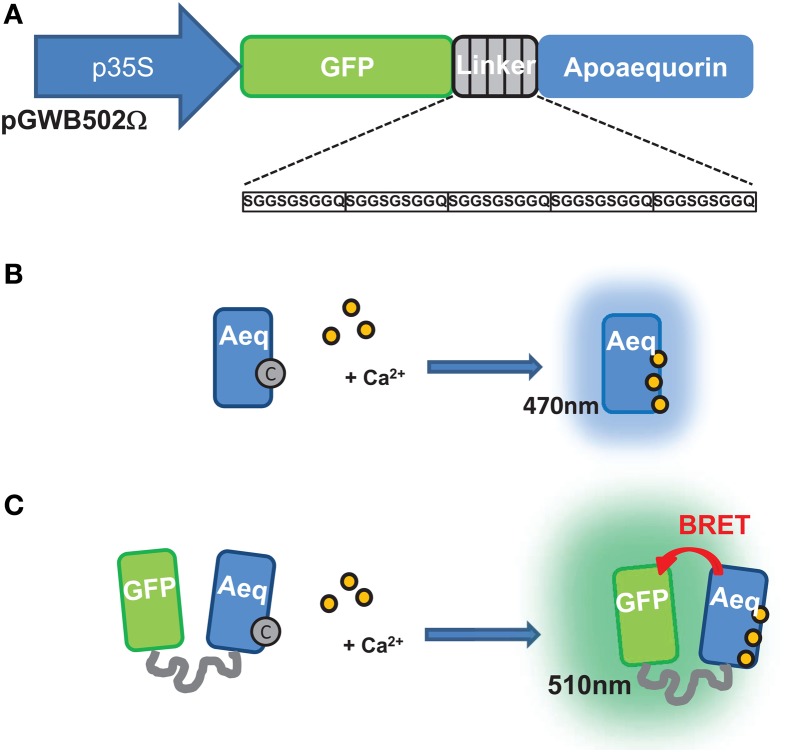
**Construction and operating principle of the G5A Ca^2+^ reporter**. **(A)** Structure of the chimeric gene encoding the G5A sensor. The open-reading frame (*ORF*) encoding the green fluorescent protein (GFP) is linked to the *ORF* encoding the apo-aequorin by five repeats of a short sequence encoding a SGGSGSGGQ oligopeptide. The structure and length of this linker ensures efficient bioluminescence resonance energy transfer (BRET) between the aequorin and the GFP (Baubet et al., 2000). Using the pGWB502Ω vector, constitutive G5A expression is driven by a 35S promoter. **(B)** Principle of the aequorin bioluminescence emission upon binding of Ca^2+^. In the presence of the coelenterazine cofactor (“C”), the apo-aequorin is reconstituted as a functional aequorin. Upon binding of three Ca^2+^ ions, the cofactor is released with emission of light at ~470 nm. **(C)** Principle of G5A fluorescence emission upon binding of Ca^2+^. Thanks to the tight molecular coupling between the aequorin and GFP moieties of G5A, a BRET phenomenon between aequorin and GFP leads to excitation of and fluorescence emission at 510 nm by the latter.

Calcium (Ca^2+^) has long been established as a second messenger. Transgenic expression of fluorescence resonance energy transfer (FRET)-based fluorescent Ca^2+^ reporters such as the popular yellow cameleon (YC) or of the bioluminescent aequorin has permitted non-invasive monitoring of free Ca^2+^ levels and enabled real-time imaging of Ca^2+^ levels in different cell-types and organisms, including plants (Knight et al., 1991; Perez Koldenkova and Nagai, 2013). The YC has been used extensively for imaging Ca^2+^ signals in specific plant cell types such as guard cells (Allen et al., 1999), germinating pollen tubes (Iwano et al., 2012), and root hairs (Miwa et al., 2006; Monshausen et al., 2008). YC is also well suitable for Ca^2+^ sensing in subcellular compartments (Krebs et al., 2012; Bonza et al., 2013). However, YC requires excitation by exogenous light, which limits its relevance in plant photosynthetic tissues due to high background emission from auto-fluorescent cell walls, chlorophyll, and secondary metabolites. Indeed, wide autofluorescent spectrum of plant leaf pigments that overlap YC emission limits visualization of changes in intensity of YC fluorescence emission upon Ca^2+^ elevation. Moreover, Ca^2+^ imaging at plant tissue level requires strong and long excitation to detect fluorescence signals. Long term Ca^2+^ measurements would result in some YC photo-bleaching and/or tissue damage, this limiting long term Ca^2+^ measurements, over 24 h for example. On the other hand, the bioluminescent Ca^2+^ reporter aequorin does not require exogenous excitation light and very little background signal is produced resulting in a high signal-to-noise ratio throughout long acquisition periods. Aequorin has the largest dynamic range among Ca^2+^ reporters, allowing the monitoring of Ca^2+^ signals over several days and over a wide range of Ca^2+^ concentrations (Alonso and Garcia-Sancho, 2011). Aequorin has been introduced into several plant species (Knight et al., 1991; Webb et al., 2010) and has enabled photon counting based monitoring of Ca^2+^ in intact plant leaves. Many reports of aequorin application in plants have been published, where photon counting with luminometers was used to describe Ca^2+^ signaling under several stress conditions. However, to image photons emitted by aequorin with good resolution in both space and time requires sophisticated detection devices such as image photon detectors (IPDs) (Webb et al., 2010) or cameras fitted with an Intensified Charge-Coupled Device (ICCD) (Webb et al., 2010) or Electron Multiplying Charge-Coupled Device (EMCCD) (Rogers et al., 2008; Webb et al., 2010). This is a significant limitation to *in planta* Ca^2+^ imaging which could be overcome by using the G5A probe, an engineered fusion between the green fluorescent protein (GFP) and aequorin (Figures 1A,C) initially developed for Ca^2+^ imaging in animal cells (Baubet et al., 2000; Rogers et al., 2005). Through a bioluminescence resonance energy transfer (BRET) from aequorin to GFP, the wavelength of the emitted photon is 510 nm, instead of 470 nm and detection yield by CCD is found optimized, compared to aequorin, with a better signal/noise ratio (Baubet et al., 2000; Rogers et al., 2005, 2008).

Here, (i) we applied G5A in Arabidopsis, (ii) we show that, in comparison to aequorin, G5A enhances *in vitro* and *in vivo* detection of weak Ca^2+^ events in intact plants, including in photosynthetic tissues and (iii) we describe a simple method that only requires a cooled-CCD camera to visualize Ca^2+^ signals in plant leaves as, for example, Ca^2+^ waves propagating along leaf veins of intact plants after imposing a salt stress to roots of these plants. It is concluded that G5A reporter is an interesting alternative to aequorin.

## Materials and methods

### Cloning G5A and engineering G5A-expressing plants

The original vector harboring the *G5A* construct (Baubet et al., 2000) was kindly provided by Dr. Philippe Brûlet's group (CNRS, Gif-sur-Yvette, France). The G5A coding sequence was cloned into the Gateway® entry vector pDONR™ by two sequential PCRs amplification using a *G5A* forward primer 5′-GGAGATAGAACC**ATGAGCAAGGGCGAGGAGCTGTTCA-3′** and a *G5A* reverse primer 5′-TCCACCTCCGG**ATCAGGGGACAGCTCCACCGTAG-**3′, followed by a second PCR using a U5 forward primer 5′-GGGGACAAGTTTGTACAAAAAAGCAGGCTTCGAAGGAGAT-AGAACCATG-3′ and a U3 reverse primer 5′-AGATTGGGGACCACTTTGTACAAGAAAGC-TGGGTCTCCACCTCCGGATC-3′. Next step was a transfer, by LR Gateway® recombination, of the *G5A* construct into the expression vector pGWB502Ω (Nakagawa et al., 2007).

**Figure 2:**
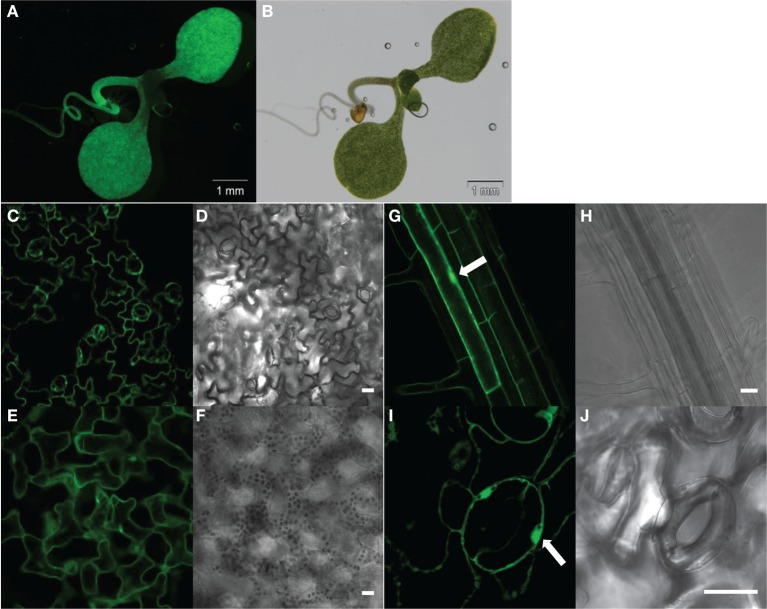
**Constitutive expression of the G5A fusion protein in all tissues of *Arabidopsis thaliana* seedlings**. Seven **(A–F)** or 21 day-old **(G–J)** plants of the *G5A* line were checked for reporter expression by excitation of GFP (at λ_ex_ = 488 nm) under a stereo microscope **(A,B)** or a confocal microscope **(C–J)**. Fluorescence emission by GFP is shown in **(A,C,E,G,I)** and corresponding bright field images are shown respectively in **(B,D,F,H,J)**. Fluorescent stereo microscope observation of intact seedlings allowed detection of GFP signals in cotyledons and the primary root **(A,B)**. Confocal microscopy observation of leaf epidermal cells **(C,D)** and mesophyll cells **(E,F)** showed good expression of G5A in leaf tissues. G5A fluorescence was observed in both the cytosol and the nucleus (arrows) of root cells **(G,H)** and of stomatal guard cells **(I,J)**. Scale bar = 20 μm.

The pGWB502Ω-*G5A* construct was introduced in *Agrobacterium tumefaciens* (GV3101), for transformation of *Arabidopsis thaliana* ecotype Col-0 by the floral dip method (Clough and Bent, 1998). G5A expressing transgenic plants were selected using hygromycin selective media and checked for GFP fluorescence emission under direct excitation of GFP at 488 nm (see Figure 2). Homozygous G5A expressing T3 and T4 plants (here below denoted *G5A* plants) were used and compared to transgenic plants expressing aequorin in the cytoplasm (Col-0 ecotype, denoted below *Aeq* plants) obtained from Prof. Marc Knight (Durham, UK).

### Plant material and growth conditions

Seeds from *Aeq* and *G5A* plants were surface-sterilized and placed on half-strength Murashige and Skoog plate medium supplemented with sucrose 1% (w/v) and with hygromycin (15 μg/mL)(*G5A*) or kanamycin (50 μg/mL)(*Aeq*), and stratified at 4°C for 2 days in the dark. Seedlings were subsequently grown in a growth chamber at 22°C with a 70% relative humidity, in long-day conditions (150 μE/m^2^/s light for 16 h a day) for 7 days. These 7-day old seedlings were either used directly or further grown in soil under short day conditions (200 μE/m^2^/s light for 8 h a day) for 3–7 weeks, as indicated.

### Luminescence measurement and imaging

#### Seedlings

*In vivo* reconstitution of functional aequorin was performed by incubating 7 day-old seedlings for 4 h at 22°C in the dark with a 2.5 μM aqueous solution of coelenterazine HCP (Interchim). For imaging, coelenterazine-treated seedlings were placed within a dark chamber over a gelosed layer (water with 1% agar) in large Petri dishes. A cooled-CCD camera (Hamamatsu 4880-30), fitted at the top of the chamber, collected photons. Sequential image acquisition was carried out using the Hipic 5.1.0 software with an exposure time per image in the 15–60 s range (as indicated in the Figure legends). Images were analyzed in ImageJ (Schneider et al., 2012). The first 5 min in each sequence were discarded because of chlorophyll autofluorescence decay.

#### Older soil-grown plants

At the end of the day, the soil was gently removed from the roots and the whole plants were incubated for 4 h at 22°C in the dark in a 2.5 μM aqueous solution of coelenterazine HCP. Plants were then placed with the roots in Qualibact® (CEB) tubes filled with water through a hole in the cap of the tube to separate leaves from roots. Plants were left for 1 h in the dark at room temperature for recovery. Images of the rosette were acquired as described above for seedlings, with a 30 s integration time. NaCl at a final concentration of 200 mM was injected with a remotely controlled syringe at the root level after 25 min of acquisition.

#### Excised mature leaves

Mature leaves were excised from 6–7-week old plants grown as described above and incubated at 22°C in the dark for 4 h in a 2.5 μM aqueous solution of coelenterazine HCP. The treated excised leaf was then transferred to a dark chamber under the cooled-CCD camera for G5A imaging as described above with 15 s exposure time by frame. For salt stress, 100 μL of 200 mM NaCl was pipetted onto the excised leaf petiole (see Figure 8A arrow) and subsequent light emission was acquired.

### Image analysis

All images were analyzed in ImageJ. Shading correction of all images was performed by subtraction of a dark field image (acquisition without sample) acquired with the same exposure time. Backgrounds of each image were normalized by subtraction of ROIs of non-plant pixels. ROIs of plant pixels were then quantified and the average values are plotted over the time. Background noise after chlorophyll fluorescence decay was determined by imaging light emission from wild-type plant leaves and then subtracted for data obtained from *G5A* or *Aeq* plants. Ca^2+^ signal velocity was determined with the MtrackJ plugin of ImageJ and the localization (x,y) of each velocity value was plotted with Matlab® software (R2006a).

### Aequorin immunoblotting

Soluble protein was extracted from 50 pooled seedlings (Mithofer and Mazars, 2002) and separated by SDS-PAGE. Immunoblotting was carried out using an anti-aequorin rabbit polyclonal antibody (Novus Biological, NB100-1877) as described by the manufacturer. Signals of immunodetection were acquired with LAS-3000 imager (Fujifilm) and quantified with Multi-Gauge v3.2 (Fujifilm).

### Calibration of the two probes G5A and aequorin

**Figure 3:**
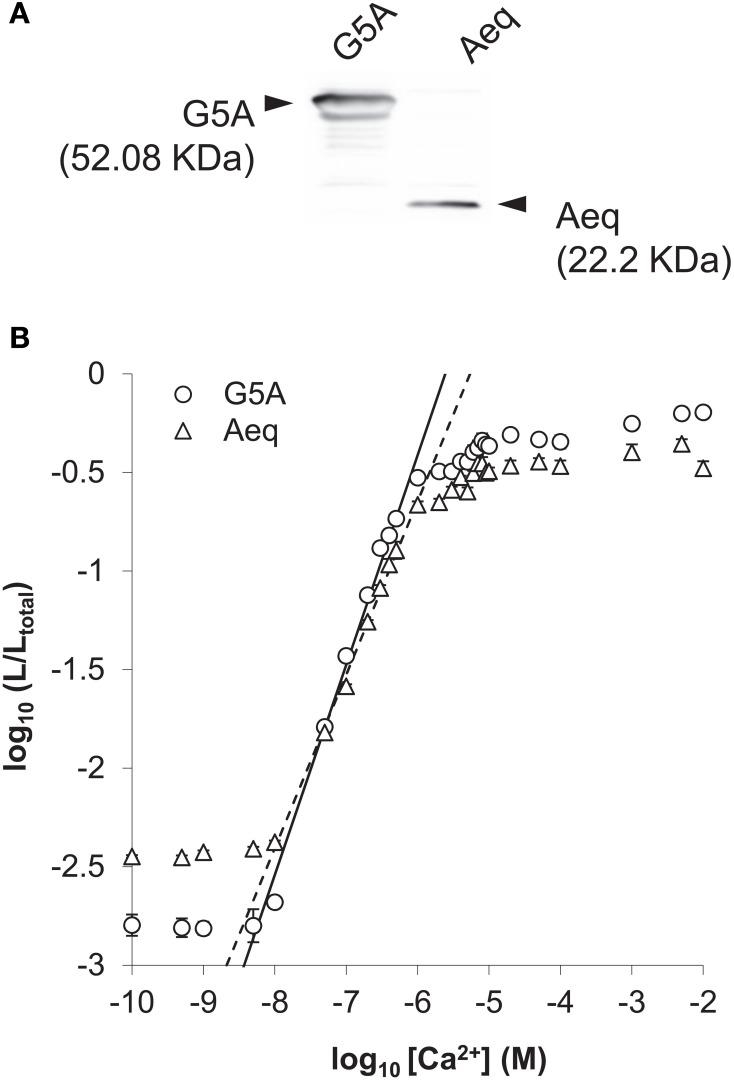
***In vitro* calibration of G5A and aequorin as Ca^2+^ reporters**. **(A)** Immunoblotting of soluble protein fractions from fifty 14 day-old plants of the *G5A* and *Aeq* lines with an anti-aequorin polyclonal antibody. Signals of immunoblotting were quantified and indicated as a G5A/aequorin ratio of protein accumulation of 1.48 ± 0.14 (average of one hundred seedlings obtained in two independent experiments ± SE, *n* = 2). **(B)** *In vitro* calibration curves of G5A and aequorin in soluble protein extracts from *G5A* and *Aeq* plants. Equivalent amounts of Ca^2+^ reporters were used for the comparison (based on assay reported in **A**) and incubated in a buffer containing 2.5 μM coelenterazine HCP cofactor for 2 h in the dark at 4°C prior light emission assay in the presence of various free Ca^2+^ amounts (see Materials and Methods). Results are expressed as the ratio ± SE (*n* = 6 in two independent experiments) of maximum light over total light (L/L_total_). The linear range of L/L_total_ ratio as a function of free Ca^2+^ concentration is shown for G5A (full line) and aequorin (dashed line).

Soluble proteins were extracted from *G5A* and *Aeq* plants as described by Mithofer and Mazars (2002) and were diluted in a buffer (Tris-HCl 200 mM, pH 7.4, EGTA 5 mM, NaCl 0.5 M, β-mercaptoethanol 5 mM) containing the coelenterazine HCP cofactor for 2 h in the dark at 4°C. Relative amounts of G5A and aequorin reporters in these crude extracts were estimated by immunoblotting (Figure 3A). So-called *G5A* and *Aeq* buffers were prepared by diluting soluble protein crude extracts from *G5A* and *Aeq* plants in Tris-HCl 200 mM, pH 7.0, EGTA 5 mM at a protein content of 0.1 μg/μL and 0.15 μg/μL respectively (to ensure that subsequent *in vitro* comparison of both reporters was made with equal quantities of them). Wells of 96-well plates were filled with 50 μL of different Ca^2+^ solutions, of which the free Ca^2+^ concentration was estimated by MaxChelator Software (http://www.stanford.edu/~cpatton/downloads.htm). To start probe calibration, 50 μL of either *G5A* or *Aeq* buffer was dispensed into each well and maximum light emitted per second (L) was measured. In a second step, 100 μL of a 2 M CaCl_2_ solution was dispensed into each well for discharging the remaining reconstituted G5A or aequorin reporters. Light emitted at this time (L_total_) allowed the total amount of functional Ca^2+^ reporter to be estimated. All light measurements were made with a plate-spectrophotometer Victor^2^ (Perkin Elmer). Collected photons were integrated over 1 s lapses during 180 s. Results are expressed as the ratio ±SE of maximum light over total light (L/L_total_).

## Results

Effective transformation using the pGWB502Ω-*G5A* construct was expected to yield a broad constitutive expression pattern of the G5A probe. This was checked in a Ca^2+^-independent manner by direct excitation, at 488 nm, of the GFP moiety of the chimera probe (Figure 2).

A strong ubiquitous GFP signal was observed in 7 day-old seedlings. The subcellular pattern of the GFP signal suggested cytosolic and nuclear localization (arrows in Figures 2G,I).

An anti-aequorin polyclonal antibody (Novus Biological, USA) was used to evaluate the amount of G5A and aequorin proteins in the soluble protein fraction (Figure 3A). This antibody revealed strong bands at 22 kDa and 52 kDa in both protein extracts from *Aeq* and *G5A* plants. The G5A/aequorin ratio (protein level) was estimated at 1.48 ± 0.14.

Calibration curves were performed with soluble protein extracts from *G5A* or *Aeq* plants. Equal amounts of G5A and aequorin reporters were used for *in vitro* calibration curves. Data are expressed as maximum light emitted per second (denoted “L”) (Fricker et al., 1999) over total light (denoted “L_total_”) ratio (Figure 3B see “Methods” section). G5A and aequorin showed similar responses to free Ca^2+^ concentration and calibration curves in Figure 3B do not differ significantly over the 10^−8^ to 10^−6^ M free Ca^2+^ range. Linear regression between 10^−8^ to 10^−6^ M free Ca^2+^ reveals a straight line with a slope of 1.065 ± 0.016 (*R*^2^ = 0.988) and 0.888 ± 0.015 (*R*^2^ = 0.987) for G5A and aequorin respectively (Figure 3B). The reciprocal relationship, i.e., between free Ca^2+^ concentration and the rate of consumption of G5A or aequorin, can be represented by the equation:

$$-\log([Ca^{2+}])=a*-\log(\frac{L}{L_{\text{Total}}})+b$$

                                                       (Fricker et al., 1999)

Coefficients *a* and *b* in the above equation are 0.93919 and 5.61289 for G5A, and 1.13646 and 5.26608 for aequorin.

**Figure 4:**
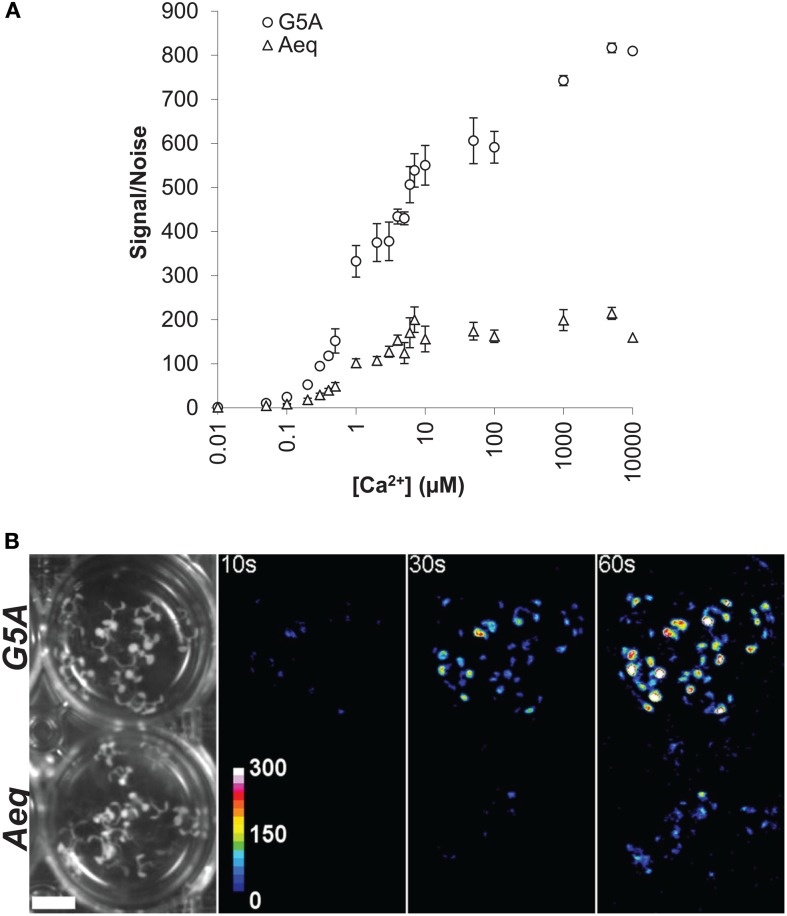
***In vitro* and *in vivo* comparison of light emission by G5A and aequorin (A)** *In vitro* assay of light emission from equal amounts of Ca^2+^ reporters G5A and aequorin is plotted against buffer free Ca^2+^ concentration. Results (mean ± SE, *n* = 6) are expressed as the ratio of signal (maximum emitted light) over noise. **(B)** Representative *in vivo* Ca^2+^ signals emitted from 7 day-old *G5A* (top) and *Aeq* (bottom) plants over different exposition time lapses (10, 30, and 60 s). Left panel shows bright field view (scale bar = 1 cm) of the imaged plants. The other panels display cumulative Ca^2+^ responses in false colors (color scale in the 10 s-labeled panel).

The bioluminescent reporter aequorin has very low noise and high signal/noise ratio (Brini, 2008; Webb et al., 2010). No signal from G5A and aequorin was detected under *in vitro conditions*, in the absence of coelenterazine HCP. In the presence of coelenterazine HCP and without Ca^2+^, G5A and aequorin noise levels were respectively 271.16 ± 10.76 and 198.67 ± 11.52 RLU (Relative Light Unit). This difference is, however, negligible compared to signal after injection of free Ca^2+^. At the basal level of cytosolic free Ca^2+^ (0.1 μM), maximum light level was increased to 6.61 × 10^3^ ± 0.32 × 10^3^ and 1.72 × 10^3^ ± 0.29 × 10^3^ RLU for G5A and aequorin respectively. At 1 μM free Ca^2+^, light levels increased up to 90.14 × 10^3^ ± 7.44 × 10^3^ and 20.25 × 10^3^ ± 1.65 × 10^3^ RLU for G5A and aequorin respectively. Data expressed as signal/noise ratio for the two reporters (Figure 4A) show that G5A is approximately 3–5 times better than aequorin. For instance, the signal/noise ratio of aequorin with 1 μM free Ca^2+^ is reached with only 300 nM free Ca^2+^ with G5A reporter.

In parallel, comparison of the two Ca^2+^ reporters was performed *in planta*. Different time lapses were tested for collecting photons emitted by *G5A* and *Aeq* plants (Figure 4B). The threshold for signal detection for basal level of free Ca^2+^ was approximately 10 s and 30 s with G5A and aequorin respectively. A 30 s time lapse allowed sufficient light to be collected from *G5A* plants while 1 min was hardly sufficient in the case of *Aeq* plants.

**Figure 5:**
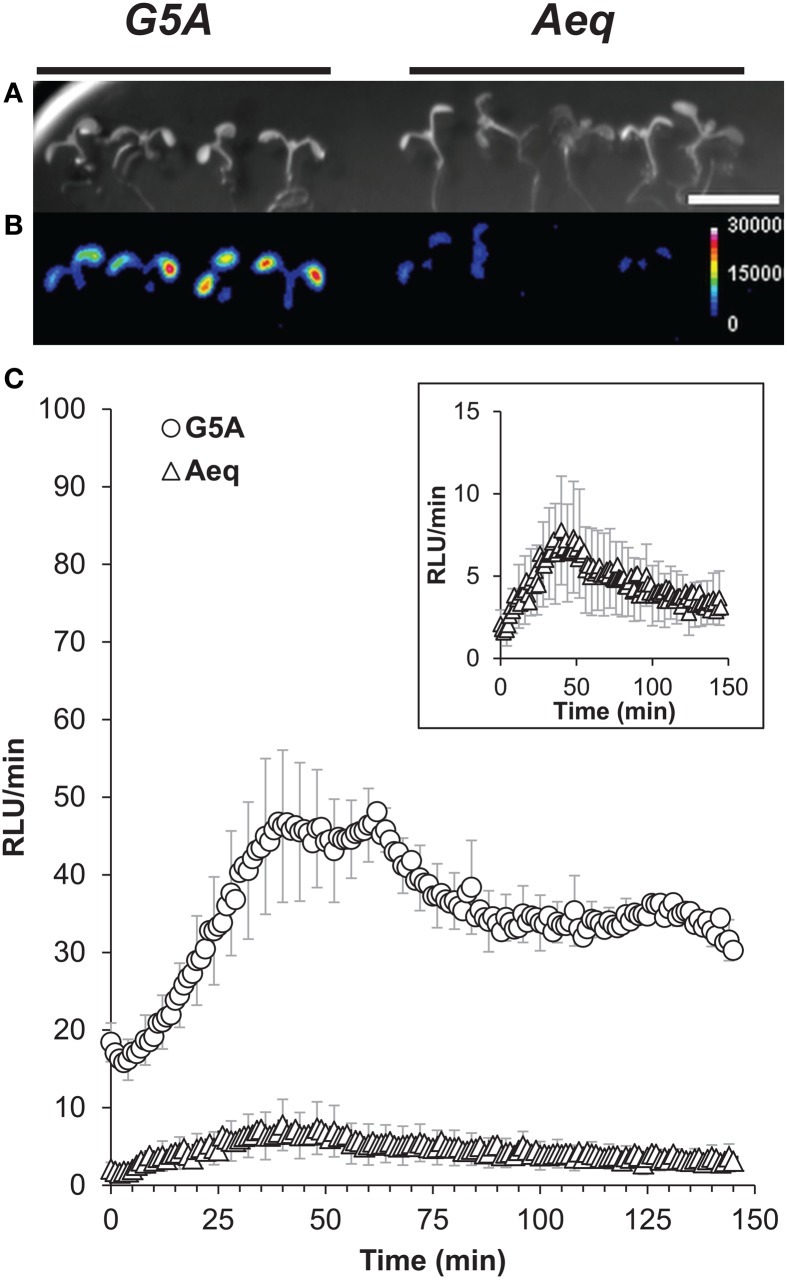
**Ca^2+^ dynamics detected in response to dark onset from plants expressing G5A or aequorin. (A)** Bright field view of batches of 7 day-old *G5A* (left) and *Aeq* (right) Arabidopsis reporter lines. Scale bar = 1 cm. **(B)** Cumulative amount of light emitted from these plants after dark onset, over 150 min, expressed in false colors (scale shown at right, in device-dependent arbitrary unit or “RLU,” standing for “relative light unit”). Representative results of 10 independent measurements of dark-induced Ca^2+^ released. **(C)** 150 min time course of the cumulative light emission over 60 s time lapses from plants in **(A**,**B)** (data are means ± SE of 10 independent experiments with four *G5A* and five *Aeq* plants). Ca^2+^ dynamics from the *Aeq* plants show kinetics (inset) similar to *G5A* plants but with approximately five times less light emission.

Sudden light-dark transition has been reported to induce weak Ca^2+^ signals in photosynthetic tissues (Johnson et al., 1995; Sai and Johnson, 2002; Dodd et al., 2006). To assess the capability of G5A to detect weak Ca^2+^ events in intact plant tissues, we challenged Arabidopsis plants with darkness: the reactions of *G5A* plants upon light-dark transition were compared to those of *Aeq* plants (Figure 5). Significantly more photons could be collected from *G5A* plants than from the *Aeq* plants over this period (Figure 5B). Successive integrations of photons over 1 min time lapses provided an overview of the Ca^2+^ signal kinetics (Figure 5C). Dark-induced Ca^2+^ signals displayed by G5A and aequorin had parallel kinetics (Figure 5C and inset), with a maximal light emission between 40 and 60 min. However, approximately five times more photons were detected from plants of the *G5A* line.

**Figure 6:**
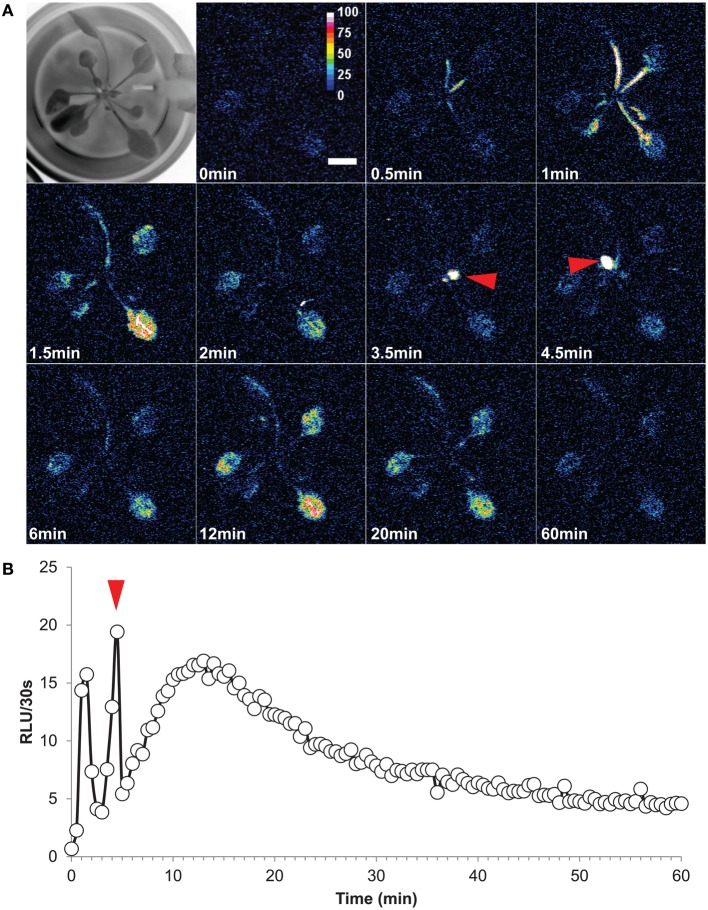
**High salt exposition of roots elicits long distance Ca^2+^ dynamics in leaves**. **(A)** Time series of images (time of capture indicated in the left bottom corner of each image) showing propagation of Ca^2+^ elevation in aerial part of an intact plant (Representative result of five independent plants). First image of the series is a bright-field view of the plant. At time = 0, a solution of NaCl (200 mM final concentration) was applied to roots and light subsequently emitted from leaves was accumulated over 60 min (each image shows cumulative light intensity over a 30 s time lapse). After 0.5 min, Ca^2+^ elevations were detected on petioles and propagation of Ca^2+^ responses to the end of each mature leaf were observed. Young leaves responded only at 3.5–4.5 min after NaCl stress (red arrows) **(B)** Quantification of light signals from the whole plant over 60 min. As in **(A)** the red arrow indicates the short Ca^2+^ response in young leaves.

This interesting G5A feature allowed us to follow the dynamics of free Ca^2+^ in leaves triggered by a salt stress applied to roots of intact plants (Figure 6 and Supplementary videos S1, S2). The stress was sensed by roots and propagated to leaves, suggesting that Ca^2+^ waves might contribute to plant adaptation to salt stress. A time series of representative results (from video S1) is presented in Figure 6A. It shows that a 30 s delay after the application of NaCl (at time = 0) was required before Ca^2+^ levels increased in the petioles. Elevated calcium levels then propagated to the rest of the leaves. It is interesting to note that the Ca^2+^ responses of mature and young leaves differed in terms of kinetics. Mature leaves responded by an initial rapid, transient, Ca^2+^ peak (Figure 6A, time = 1–2 min) followed by a second very slow, wave-like, increase and subsequent decrease of free Ca^2+^ level lasting more than 50 min (from 6 to 60 min) with a maximum at 12–13 min. The young leaves displayed a single rapid Ca^2+^ transient peak (Figure 6A red arrows), similar to that observed in mature leaves although slightly later (3.5–4.5 min after salt stress application). Defining the whole plant as an ROI and plotting the time course of the signal summed over each 30 s lapse (in RLU/30 s) for 60 min after salt stress application yielded a dynamic view of these Ca^2+^ events at leaf level (Figure 6B). It was found that both the Ca^2+^ peaks (observed for mature and young leaves) and the Ca^2+^ wave had a maximum at 19–20 RLU/30 s (Figure 6B). Despite inevitable variations from a plant to another one, an analogous pattern of distribution in space and time of Ca^2+^ events was observed when challenging a plant with a salt stress at the root level (Supplementary video S2).

**Figure 7:**
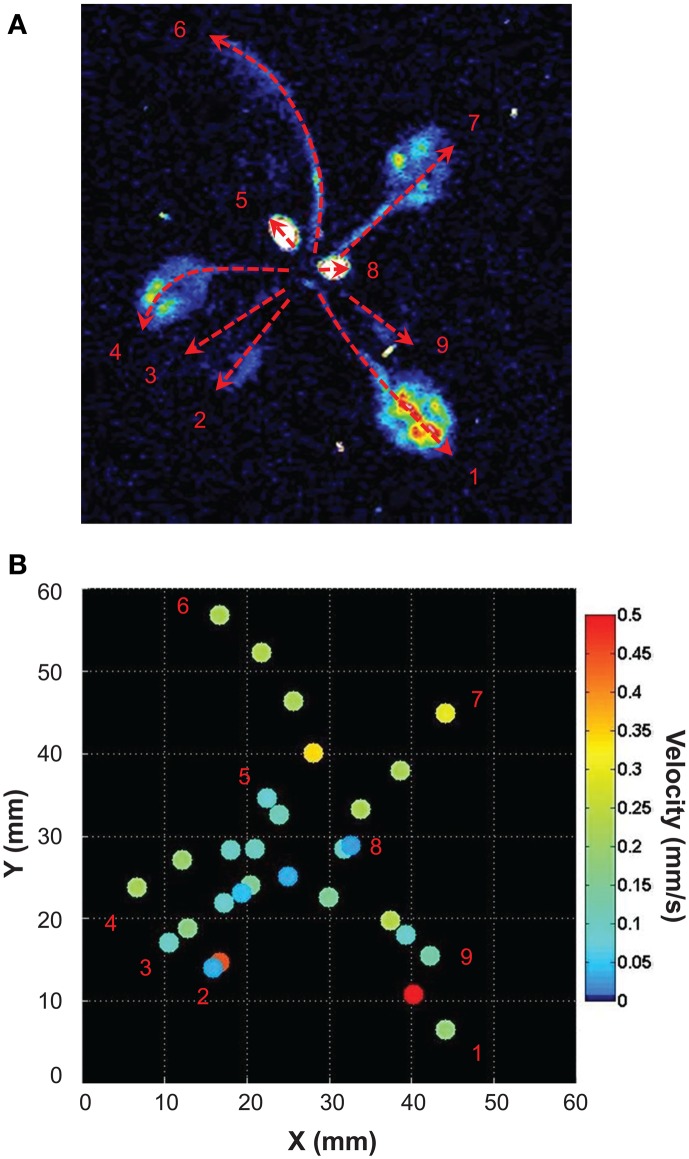
**Analysis of the propagation of Ca^2+^ elevations induced by high salt stimulus applied to roots**. **(A)** Free Ca^2+^ elevations on each leaf were analyzed with ImageJ. Velocities (mm/s) of Ca^2+^ responses were determined for each leaf along paths figured by red dashed arrows (values of Ca^2+^ response velocity are presented on Table 1). **(B)** Propagation speeds along the main leaf vain are indicated for selected points (same space scale as in **A**). False color scale is in mm/s.

Light emitted by G5A in intact plants facing a salt stress therefore appeared to be sufficiently intense to image the propagation of Ca^2+^ signals in leaves with good time resolution. We performed a simple analysis of Ca^2+^ waves on each leaf of plants subjected to a salt stress applied to roots. Ca^2+^ signal velocities were then calculated for each leaf of the plant shown in Figure 6 (red dashed arrows, Figure 7). This shows that velocity was not constant within a given leaf (it decreased at leaf tip) and differed depending on the leaf. Detailed numerical values are given (Table 1): maximum and minimum of velocities were 0.52 and 0.03 mm/s respectively.

**Table 1:** **Ca^2+^ wave properties in different leaves of an intact plant**.

**Leaf number**	**Total length (mm)**	**Velocity mean (mm/s)**	**Velocity minimum (mm/s)**	**Velocity maximum (mm/s)**	Duration ^*^ (s)	Latency ^*^ (s)
1	25.96	0.29 ± 0.10	0.15	0.52	90	30
2	15.55	0.17 ± 0.12	0.03	0.45	90	30
3	16.87	0.11 ± 0.02	0.05	0.18	150	60
4	18.50	0.15 ± 0.03	0.10	0.23	120	30
5	5.84	0.10 ± 0.01	0.08	0.11	60	240
6	30.81	0.26 ± 0.03	0.23	0.34	120	30
7	22.19	0.25 ± 0.02	0.22	0.30	90	30
8	3.68	0.06 ± 0.03	0.03	0.09	60	210
9	13.67	0.15 ± 0.04	0.09	0.24	90	90

* *Time resolution is limited by the time acquisition (30 s)*.

**Figure 8:**
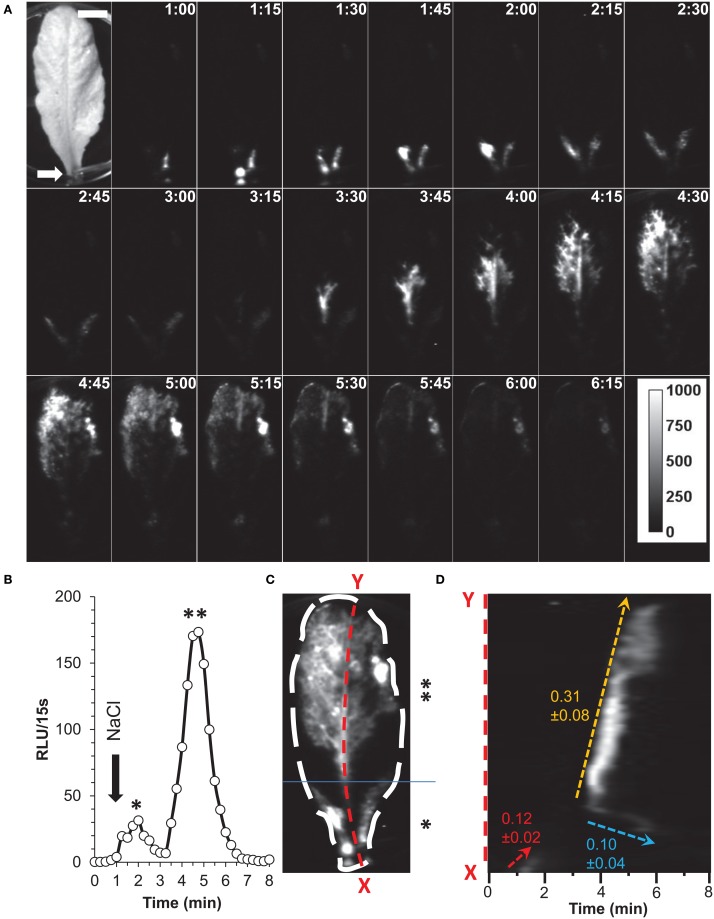
**Salt application on detached leaf elicits Ca^2+^ waves along the vascular tissue. (A)** Time series of images (time of capture indicated in the right top corner of each image in min:s) showing propagation of Ca^2+^ responses in a detached leaf. First image of the series is a bright-field view of the plant. At time = 1 min, 100 μL of a solution of NaCl (200 mM) was dispensed onto the petiole (white arrow) and light subsequently emitted from leaves was integrated over 7 min (each image shows cumulative light over a 15 s time lapse). Ca^2+^ responses were immediately detected on petioles and propagation of Ca^2+^ responses to the end of leaf was observed. Scale bar = 1 cm. Calibration bar in gray scale is shown in the last image (RLU). Representative result of five independent experiments. **(B)** Quantification of light signals from ROI for the whole leaf (see **C**) over 8 min (in RLU per 15 s). At time = 1 min, NaCl was applied onto the petiole (black arrow). The two peaks denoted by * and ** correspond to a Ca^2+^ increase in the basal third and the rest of the leaf respectively. **(C)** Localization of Ca^2+^ responses is represented by a z-stack of standard deviation of Ca^2+^ signals over the 8 min of measurement. Ca^2+^ signals seem to take place in leaf veins. The basal third of the leaf is indicated by * and the rest of the leaf by **. Line scan of primary vein (red dashed line X–Y) is analyzed in **(D)**. **(D)** Kymographic representation of Ca^2+^ signals in the primary vein (X–Y from **C**). Three different velocities of Ca^2+^ signals could be measured and are indicated.

Further applications of G5A were investigated by imposing similar salt stress on mature leaves excised from 7–8 week-old plants (Figure 8). Application of 100 μL of 200 mM NaCl onto the petiole end of an excised leaf (Figure 8A, white arrow) was enough to generate detectable elevations in free calcium after 1 min. They started immediately after the exposure to the NaCl solution at the site of application before they spread throughout the rest of the leaf (Figure 8A and Supplementary videos S3, S4). The propagation of free calcium elevation seems to be different in the basal third part of the leaf (denoted by * in Figure 8C) as compared to the rest of the leaf (denoted by ** in Figure 8C). In the early phase of response to the applied stress, increase in free Ca^2+^ were observed in the peripheral regions of the basal third part of the leaf and not in the middle vein. Subsequently, Ca^2+^ responses seem to propagate throughout the leaf, firstly, along the vascular tissues (primary and secondary veins) and later in the mesophyll tissue. Quantification of increase in free Ca^2+^ from the entire leaf shows that salt stress induced two different peaks of Ca^2+^, which correspond to these two successive episodes of Ca^2+^ increase, firstly in the basal part of the leaf, subsequently in the rest of the leaf (as indicated by asterisks in Figure 8B as described for Figure 8C).

**Figure 9:**
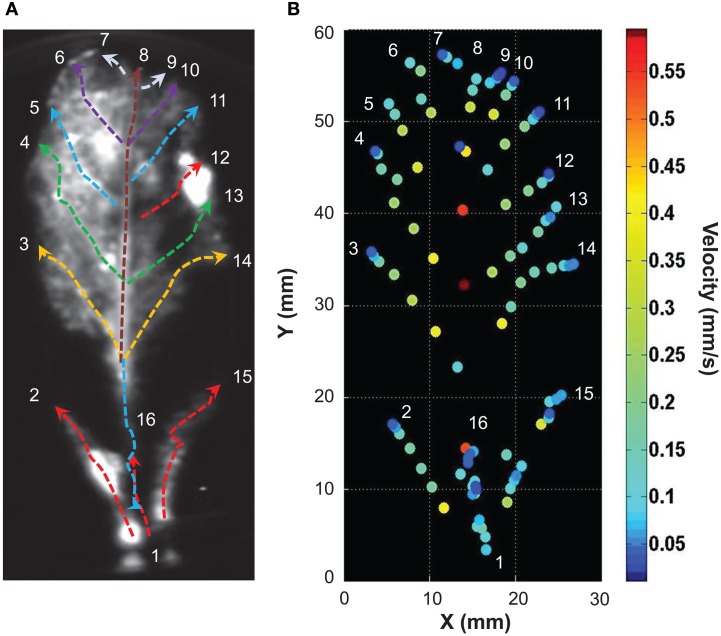
**Analysis of the propagation of calcium elevation in detached leaves induced by high salt exposure. (A)** Ca^2+^ responses on each vein were analyzed in ImageJ. Velocities (mm/s) of Ca^2+^ signals were determined along paths represented by dashed arrows (details of Ca^2+^ signal velocity, duration and latency are presented on Table 2; colors of dashed arrows refer to the latency values so that Ca^2+^ waves that started at similar times are in the same color). **(B)** Propagation speeds along the main leaf vein are indicated for selected points (same space scale as in **A**). False color scale is in mm/s.

In this example (representative of five independent leaves), elevation of free Ca^2+^ induced by NaCl needed 315 s (image at 1:00 to 6:15) to travel through a 57 mm-long leaf. Thus, in this example the average velocity was 0.181 mm/s. A further analysis was performed on the primary vein of this leaf (Figure 8C, red dashed line X–Y), a kymographic representation of velocity value on the axis X–Y shows that there were three different Ca^2+^ response velocities (Figure 8D). Two of them (red dashed and orange dashed arrows) spread acropetally (from X to Y) whereas one propagated the opposite way (Y–X, cyan dashed arrow). Interestingly, between the first third and the second third of the leaf, was observed a region where no Ca^2+^ signals were detected with G5A. Despite this gap, Ca^2+^ signal propagation was observed all along the XY axis (Figure 8C and Supplementary video S3). Analyses of Ca^2+^ waves on this excised leaf (representative of five leaves) show that Ca^2+^ signal velocities along different veins (Figure 9A dashed arrows) were different. Local velocity values were plotted on the image (as spots in false-color scale, Figure 9B). Higher velocities in the center of the leaf and slower velocities at leaf borders were found (Figure 9B). Details of velocities of Ca^2+^ signals in leaf veins, including on X–Y axis (#1, #8, and #16) are presented in Table 2 below. Despite inevitable variation from a leaf excised from a plant to another leaf excised from another plant, the nature and pattern (both in space and time) was essentially reproducible (see Supplemental video S4).

**Table 2:** **Ca^2+^ wave properties in detached leaves upon high salt exposure**.

**Number of Ca^2+^ responses**	**Total length (mm)**	**Velocity mean (mm/s)**	**Velocity minimum (mm/s)**	**Velocity maximum (mm/s)**	Duration ^*^ (s)	Latency ^*^ (s)
1	8.62	0.12 ± 0.02	0.06	0.20	75	0
2	17.01	0.16 ± 0.04	0.03	0.41	105	30
3	17.34	0.19 ± 0.05	0.04	0.39	90	150
4	20.33	0.19 ± 0.04	0.03	0.40	105	165
5	13.18	0.22 ± 0.06	0.09	0.36	60	195
6	10.63	0.18 ± 0.04	0.10	0.28	60	195
7	2.88	0.06 ± 0.02	0.04	0.10	45	255
8	32.90	0.31 ± 0.08	0.09	0.60	105	150
9	2.78	0.04 ± 0.01	0.01	0.07	75	240
10	9.94	0.13 ± 0.06	0.02	0.35	75	195
11	10.21	0.11 ± 0.03	0.03	0.23	90	195
12	9.77	0.13 ± 0.04	0.02	0.24	75	195
13	14.41	0.14 ± 0.03	0.04	0.27	105	165
14	17.59	0.15 ± 0.04	0.04	0.40	120	150
15	20.04	0.11 ± 0.03	0.03	0.35	180	0
16	20.25	0.10 ± 0.04	0.02	0.54	195	150

* *Time resolution is limited by the time acquisition (15 s)*.

## Discussion

Since the successful use of aequorin as a Ca^2+^-signaling reporter in plant tissues (Knight et al., 1991), examples of aequorin imaging in plants have relied on the use of ultra-sensitive camera devices (intensified-CCD or electron-multiplying-CCD) to detect the few photons emitted by aequorin. They have revealed, for example, that Ca^2+^ oscillations occur during diurnal rhythms in plant leaves (Johnson et al., 1995; Sai and Johnson, 2002; Dodd et al., 2006). Optimized imaging of aequorin signals in plants has been reported recently that, with integration time down to 40 s, showed stimulus- and tissue-specific Ca^2+^ signatures in seedlings (Zhu et al., 2013). In these examples of aequorin imaging in plants, however, low light detection relied upon sophisticated and costly equipment. Another interesting development of aequorin-based approach to *in planta* Ca^2+^ signaling has recently been reported: expression of aequorin in specific cell types of Arabidopsis was used to determine which cell types release calcium into the cytosol in response to a given stimulus (Marti et al., 2013). Photometry (with high time resolution) of aequorin emission from plants expressing the Ca^2+^-sensor in specific leaf cell–types (mesophyll cells, guard cells, [peri-]vascular cells, epidermal cells, and trichomes) may be used to follow various stresses. These experimental conditions allowed the collected information to be ascribed to a given cell-type, without spatial localization of the measured signals.

The G5A-based method we report here provides an interesting complement to these recent improvements of aequorin-based methods. In the *Aequoria victoria* jellyfish, a naturally evolved BRET phenomenon between aequorin and GFP occurs. Several artificial proteins assembling aequorin with GFP-derived proteins have been engineered to mimic the natural BRET observed in *Aequoria victoria*. Performance of these artificial Ca^2+^ reporters depends on the linker motif placed in between the BRET partner proteins (Baubet et al., 2000; Gorokhovatsky et al., 2004). Comparison of different linkers in GFP-aequorin protein fusions has demonstrated that the five repeat motifs used here in the so-called G5A artificial reporter (see Figure 1) allows a high BRET efficiency (Baubet et al., 2000). Subsequently, the G5A reporter has been successfully used to monitor Ca^2+^ elevation at cellular and organ levels in animals (Baubet et al., 2000; Chiesa et al., 2001; Cassidy and Radda, 2005; Rogers et al., 2005, 2008; Martin et al., 2007; Naumann et al., 2010). To date, however, no G5A application in plants has been reported.

Although our *in vitro* assays show similar calibration curves for both reporters (Figure 3B), there was a significantly better signal/noise ratio for G5A than for aequorin (Figure 4A) corresponding to a 3–5 fold increase in light collected from the former compared to the latter. Such an amplification is consistent with the data obtained *in planta* (Figures 4B, 5) where small changes of free Ca^2+^ concentration were more easily detected with G5A than with aequorin. The (up to) 5-fold amplification of light in plants expressing G5A is similar to the 5.7-fold increase in the light signal resulting from BRET between luciferin and GFP in *Renilla reniformis* (Ward and Cormier, 1976). It is interesting to note however that the improved detection of light emission from aequorin through BRET is still not completely understood (Webb et al., 2010). In the jellyfish *Aequorea victoria*, aequorin is associated with the GFP that allows the amplification of light by BRET phenomena. *In vitro*, the binding between aequorin and GFP does not occur, even at high concentration and no BRET events was observed (Baubet et al., 2000). Fusion of aequorin and GFP is a prerequisite to observe such natural light amplification seen in jellyfish. Baubet et al. have designed an optimized linker that results in sufficient high quantum yield to visualize Ca^2+^ induced light emission from G5A (Baubet et al., 2000).

An issue for plant cell physiologists is the autofluorescence of chlorophyll and other organic pigments which hinders the use of fluorescent probes with overlapping spectroscopic properties. For instance chlorophyll *b* and carotenoids (lutein, neoxanthin, and violaxanthin) have major absorption peaks between 469 and 490 nm (Rivadossi et al., 2004; Taylor et al., 2006) and may absorb photons emitted at ~470 nm by aequorin in leaf cells. As a consequence of the efficient BRET between its aequorin and GFP moieties, G5A emits photons at~510 nm (Baubet et al., 2000; Rogers et al., 2005, 2008), thus reducing absorbance by plant tissues and resulting in better detection by cameras or luminometers. This also could contribute to the better performance of G5A compared to aequorin in the present *in planta* experiments (Figure 5C).

In addition to these considerations, the G5A-based results that we report here exemplify the possibilities that this Ca^2+^ reporter holds for plant biologists. Comparison of *Aeq* and *G5A* plants subjected to darkness shows that similar responses could be visualized with both Ca^2+^ reporters, but five times more light was emitted from *G5A* plants (Figure 5). This suggests that G5A is an alternative tool to aequorin for Ca^2+^ imaging when signals are either too low or if ultra-sensitive camera is not available. Moreover, further examples of potential application of G5A are introduced here, showing the analysis of long-distance Ca^2+^ signaling (potentially involved in the coordination of the integrative responses of plant to stresses, here salt stress, Figures 6–9).

Long distance propagation of Ca^2+^ signals (“Ca^2+^ waves”) is attracting increasing interest in the plant biology community (Steinhorst and Kudla, 2013) and in this context, G5A seems to be a complementary tool, along with fluorescent Ca^2+^ reporters, to investigate complex cell to cell communication within plants. Most of Ca^2+^ imaging experiments in intact tissue with a fluorescent reporter have been carried out in roots (Fasano et al., 2001; Monshausen et al., 2011; Gjetting et al., 2012), where auto-fluorescence is much less of an obstacle than in leaves. It is by using FRET-confocal laser scanning microscopy that Ca^2+^ imaging on leaves with YC has recently been reported (Benikhlef et al., 2013; Verrillo et al., 2014). Aequorin imaging requires ultra-sensitive cameras (intensified-CCD or electron-multiplied-CCD) while G5A imaging does not. The high dynamics and intensity of G5A light emission upon Ca^2+^ events open opportunities to detect low Ca^2+^ signals and to analyse their propagation with good time resolution, using a “regular” cooled CCD camera. In practical terms, time resolution is the time required to make an image (a frame) with an acceptably high signal/noise ratio. Under the present experimental conditions, integration time for a frame was well under 1 min: depending on amplitude of the Ca^2+^ signal this integration time ranged from 30 s (Figure 6) down to 15 s (Figure 8) for whole seedlings or mature leaves and even down to 5 s in the most favorable case (wounding stress, data not shown). Thus, values of Ca^2+^ wave propagation speed as fast as 0.5–0.6 mm/s can be resolved (Figure 9, Tables 1, 2).

We consider that G5A reporter opens exciting perspectives for the study of cell-to-cell communication in plants. The physiological meaning of Ca^2+^ waves observed within vascular tissues (but not solely there) is one of the interesting aspects which could be investigated. Velocity values observed for these Ca^2+^ signals are of the same order of magnitude than those of “fast” electrical signals (i.e., “action potentials”) reported to travel leaf tissues in Arabidopsis (Favre et al., 2011). This substantiates the hypothesis of an interplay between Ca^2+^ and electrical signaling (Król et al., 2011). Recently, glutamate receptor-like putative Ca^2+^ channels were reported to play a role in leaf to leaf signaling after wounding (Mousavi et al., 2013), together with electrical signals. In this context, combining G5A-based imaging of Ca^2+^ with electrophysiological recording of electrical signals might be a powerful method to decipher the molecular basis of electrical signaling in plants upon different types of stress, including wounding.

In conclusion, G5A allows to image free Ca^2+^ elevation in intact plant leaves, making this probe a promising addition in the toolbox of plant cell physiology. Ca^2+^ imaging in intact plant leaves with widely affordable imaging equipment has the potential to boost the investigation of Ca^2+^ signaling in plants.

### Conflict of interest statement

The authors declare that the research was conducted in the absence of any commercial or financial relationships that could be construed as a potential conflict of interest.

## Supplementary material

Supplementary video S1 **Light emission from 4 week-old G5A-expressing leaves upon a high salt stimulus applied to roots of the plant presented in Figures 5, 6**. The left part **(A)** shows the bright field view of the plant (scale bar is 2 cm). Video in the right part **(B)** is made of frames, each corresponding to photons collected over a 30 s time lapse, displayed at a rate of three frames per second (video rate is ×90). At time zero of the video, the plant has been adapted to dark for 20 min and no Ca^2+^ signals were detected (data not shown). Application of 200 mM NaCl to roots at *t* = 5 min (3.33 s, video time) induced propagation of Ca^2+^ elevation over long distances from roots to leaves over 77 min. NaCl-induced Ca^2+^ waves were different in expanded leaves and in young ones. Propagation of strong Ca^2+^ elevations to fully expanded leaves can be visualized just after the NaCl stress and is followed by slow Ca^2+^ variations in leaves over 77 min. Small young leaves emit strong and transient Ca^2+^ elevation only 3.5–4.5 min after the NaCl stress (5.66–6.66 s, video time).

Supplementary video S2 **Second example of light emission from 4 week-old G5A-expressing leaves upon a high salt stimulus applied to roots of the same plant**. The left part **(A)** shows the bright field view of this plant (scale bar is 2 cm). Video in the right part **(B)** is made of frames, each corresponding to photons collected over a 30 s time lapse, displayed at a rate of three frames per second (video rate is ×90). At time zero of the video, the plant has been adapted to dark for 20 min and no Ca^2+^ signals were detected (data not shown). Application of 200 mM NaCl to roots at *t* = 5 min (3.33 s, video time) induced propagation of a Ca^2+^ elevation over long distances from roots to leaves over 150 min. As in video S1, NaCl-induced Ca^2+^ waves were different in expanded leaves and in young ones. In this other plant, however, propagation rates and kinetics of Ca^2+^ changes were different from those in the plant featured in video S1, exemplifying the complex nature of Ca^2+^ responses in intact plants.

Supplementary video S3 **Light emission by a leaf detached from a *G5A* plant upon a high-salt stimulus applied to the petiole**. The left part **(A)** shows the bright field view of the leaf (scale bar is 1 cm). Video in the right part **(B)** is made of frames, each corresponding to photons collected over a 15 s time lapse, displayed at a rate of six frames per second (video rate is ×90). At time zero no light is detected. Application of 200 mM NaCl to petiole at *t* = 1 min (0.66 s, video time) induced Ca^2+^ waves from petiole to leaf tips over 7 min.

Supplementary video S4 **Second example of light emission from a detached G5A-expressing leaf upon a high salt stimulus applied to the petiole**. The left part **(A)** shows the bright field view of the leaf (scale bar is 1 cm). Video in the right part **(B)** is made of frames, each corresponding to photons collected over a 15 s time lapse, displayed at a rate of six frames per second (video rate is ×90). At time zero no light is detected. Application of 200 mM NaCl to petiole at *t* = 1 min (0.66 s, video time) induced Ca^2+^ waves from the petiole to leaf tips over 17 min. In this leaf from another plant, however, propagation rates and kinetics of Ca^2+^ changes were different from those in the leaf featured in video S3, exemplifying the complex nature of Ca^2+^ responses in excised leaves.

## References

[B1] Allen G. J. Kwak J. M. Chu S. P. Llopis J. Tsien R. Y. Harper J. F. (1999). Cameleon calcium indicator reports cytoplasmic calcium dynamics in Arabidopsis guard cells. Plant J. 19, 735–747 10.1046/j.1365-313x.1999.00574.x 10571859

[B2] Alonso M. T. Garcia-Sancho J. (2011). Nuclear Ca^2+^ signalling. Cell Calcium 49, 280–289 10.1016/j.ceca.2010.11.004 21146212

[B3] Baubet V. Le Mouellic H. Campbell A. K. Lucas-Meunier E. Fossier P. Brulet P. (2000). Chimeric green fluorescent protein-aequorin as bioluminescent Ca^2+^ reporters at the single-cell level. Proc. Natl. Acad. Sci. U.S.A. 97, 7260–7265 10.1073/pnas.97.13.7260 10860991 PMC16533

[B4] Benikhlef L. L'Haridon F. Abou-Mansour E. Serrano M. Binda M. Costa A. (2013). Perception of soft mechanical stress in Arabidopsis leaves activates disease resistance. BMC Plant Biol. 13:133 10.1186/1471-2229-13-133 24033927 PMC3848705

[B5] Bonza M. C. Loro G. Behera S. Wong A. Kudla J. Costa A. (2013). Analyses of Ca^2+^ accumulation and dynamics in the endoplasmic reticulum of Arabidopsis root cells using a genetically encoded Cameleon sensor. Plant Physiol. 163, 1230–1241 10.1104/pp.113.226050 24082028 PMC3813646

[B6] Brini M. (2008). Calcium-sensitive photoproteins. Methods 46, 160–166 10.1016/j.ymeth.2008.09.011 18848993

[B7] Cassidy P. J. Radda G. K. (2005). Molecular imaging perspectives. J. R. Soc. Interface 2, 133–144 10.1098/rsif.2005.0040 16849174 PMC1629073

[B8] Chiesa A. Rapizzi E. Tosello V. Pinton P. De Virgilio M. Fogarty K. E. (2001). Recombinant aequorin and green fluorescent protein as valuable tools in the study of cell signalling. Biochem. J. 355, 1–12 10.1042/0264-6021:3550001 11256942 PMC1221705

[B9] Clough S. J. Bent A. F. (1998). Floral dip: a simplified method for Agrobacterium-mediated transformation of *Arabidopsis thaliana*. Plant J. 16, 735–743 10.1046/j.1365-313x.1998.00343.x 10069079

[B10] Dodd A. N. Jakobsen M. K. Baker A. J. Telzerow A. Hou S. W. Laplaze L. (2006). Time of day modulates low-temperature Ca^2+^ signals in Arabidopsis. Plant J. 48, 962–973 10.1111/j.1365-313X.2006.02933.x 17227550

[B11] Fasano J. M. Swanson S. J. Blancaflor E. B. Dowd P. E. Kao T. H. Gilroy S. (2001). Changes in root cap pH are required for the gravity response of the Arabidopsis root. Plant Cell 13, 907–921 10.2307/3871348 11283344 PMC135544

[B12] Favre P. Greppin H. Degli Agosti R. (2011). Accession-dependent action potentials in Arabidopsis. J. Plant Physiol. 168, 653–660 10.1016/j.jplph.2010.09.014 21112666

[B13] Fricker M. D. Plieth C. Knight H. Blancaflor E. Knight M. R. White N. S. (1999). Chapter forty-two - fluorescence and luminescence techniques to probe ion activities in living plant cells, in Fluorescent and Luminescent Probes for Biological Activity, 2nd Edn, ed Mason W. T. (London: Academic Press), 569–596

[B14] Gjetting K. S. Ytting C. K. Schulz A. Fuglsang A. T. (2012). Live imaging of intra- and extracellular pH in plants using pHusion, a novel genetically encoded biosensor. J. Exp. Bot. 63, 3207–3218 10.1093/jxb/ers040 22407646 PMC3350929

[B15] Gorokhovatsky A. Y. Marchenkov V. V. Rudenko N. V. Ivashina T. V. Ksenzenko V. N. Burkhardt N. (2004). Fusion of Aequorea victoria GFP and aequorin provides their Ca^2+^-induced interaction that results in red shift of GFP absorption and efficient bioluminescence energy transfer. Biochem. Biophys. Res. Commun. 320, 703–711 10.1016/j.bbrc.2004.06.014 15240105

[B16] Iwano M. Ngo Q. A. Entani T. Shiba H. Nagai T. Miyawaki A. (2012). Cytoplasmic Ca^2+^ changes dynamically during the interaction of the pollen tube with synergid cells. Development 139, 4202–4209 10.1242/dev.081208 23093426

[B17] Johnson C. H. Knight M. R. Kondo T. Masson P. Sedbrook J. Haley A. (1995). Circadian oscillations of cytosolic and chloroplastic free calcium in plants. Science 269, 1863–1865 10.1126/science.7569925 7569925

[B18] Knight M. R. Campbell A. K. Smith S. M. Trewavas A. J. (1991). Transgenic plant aequorin reports the effects of touch and cold-shock and elicitors on cytoplasmic calcium. Nature 352, 524–526 10.1038/352524a0 1865907

[B19] Krebs M. Held K. Binder A. Hashimoto K. Den Herder G. Parniske M. (2012). FRET-based genetically encoded sensors allow high-resolution live cell imaging of Ca^2+^ dynamics. Plant J. 69, 181–192 10.1111/j.1365-313X.2011.04780.x 21910770

[B41] Król E. Płachno B. J. Adamec L. Stolarz M. Dziubińska H. Trębacz K. (2011). Quite a few reasons for calling carnivores ‘the most wonderful plants in the world’. Ann. Bot. 109, 47–64 10.1093/aob/mcr249 21937485 PMC3241575

[B20] Marti M. C. Stancombe M. A. Webb A. A. (2013). Cell- and stimulus type-specific intracellular free Ca^2+^ signals in Arabidopsis. Plant Physiol. 163, 625–634 10.1104/pp.113.222901 24027243 PMC3793043

[B21] Martin J. R. Rogers K. L. Chagneau C. Brulet P. (2007). *In vivo* bioluminescence imaging of Ca^2+^ signalling in the brain of Drosophila. PLoS ONE 2:e275 10.1371/journal.pone.0000275 17342209 PMC1803028

[B22] Mithofer A. Mazars C. (2002). Aequorin-based measurements of intracellular Ca^2+^-signatures in plant cells. Biol. Proced. Online 4, 105–118 10.1251/bpo40 12734562 PMC145563

[B23] Miwa H. Sun J. Oldroyd G. E. Downie J. A. (2006). Analysis of calcium spiking using a cameleon calcium sensor reveals that nodulation gene expression is regulated by calcium spike number and the developmental status of the cell. Plant J. 48, 883–894 10.1111/j.1365-313X.2006.02926.x 17227545

[B24] Monshausen G. B. Messerli M. A. Gilroy S. (2008). Imaging of the Yellow Cameleon 3.6 indicator reveals that elevations in cytosolic Ca^2+^ follow oscillating increases in growth in root hairs of Arabidopsis. Plant Physiol. 147, 1690–1698 10.1104/pp.108.123638 18583529 PMC2492656

[B25] Monshausen G. B. Miller N. D. Murphy A. S. Gilroy S. (2011). Dynamics of auxin-dependent Ca^2+^ and pH signaling in root growth revealed by integrating high-resolution imaging with automated computer vision-based analysis. Plant J. 65, 309–318 10.1111/j.1365-313X.2010.04423.x 21223394

[B26] Mousavi S. A. Chauvin A. Pascaud F. Kellenberger S. Farmer E. E. (2013). GLUTAMATE RECEPTOR-LIKE genes mediate leaf-to-leaf wound signalling. Nature 500, 422–426 10.1038/nature12478 23969459

[B27] Nakagawa T. Suzuki T. Murata S. Nakamura S. Hino T. Maeo K. (2007). Improved Gateway binary vectors: high-performance vectors for creation of fusion constructs in transgenic analysis of plants. Biosci. Biotechnol. Biochem. 71, 2095–2100 10.1271/bbb.70216 17690442

[B28] Naumann E. A. Kampff A. R. Prober D. A. Schier A. F. Engert F. (2010). Monitoring neural activity with bioluminescence during natural behavior. Nat. Neurosci. 13, 513–520 10.1038/nn.2518 20305645 PMC2846983

[B29] Perez Koldenkova V. Nagai T. (2013). Genetically encoded Ca^2+^ indicators: Properties and evaluation. Biochim. Biophys. Acta. 1833, 1787–1797 10.1016/j.bbamcr.2013.01.011 23352808

[B30] Rivadossi A. Zucchelli G. Garlaschi F. M. Jennings R. C. (2004). Light absorption by the chlorophyll a-b complexes of photosystem II in a leaf with special reference to LHCII. Photochem. Photobiol. 80, 492–498 10.1562/0031-8655(2004)080<0492:LABTCA>2.0.CO;2 15623336

[B31] Rogers K. L. Martin J. R. Renaud O. Karplus E. Nicola M. A. Nguyen M. (2008). Electron-multiplying charge-coupled detector-based bioluminescence recording of single-cell Ca^2+^. J. Biomed. Opt. 13, 031211 10.1117/1.2937236 18601535

[B32] Rogers K. L. Stinnakre J. Agulhon C. Jublot D. Shorte S. L. Kremer E. J. (2005). Visualization of local Ca^2+^ dynamics with genetically encoded bioluminescent reporters. Eur. J. Neurosci. 21, 597–610 10.1111/j.1460-9568.2005.03871.x 15733079

[B33] Sai J. Johnson C. H. (2002). Dark-stimulated calcium ion fluxes in the chloroplast stroma and cytosol. Plant Cell 14, 1279–1291 10.1105/tpc.000653 12084827 PMC150780

[B34] Schneider C. A. Rasband W. S. Eliceiri K. W. (2012). NIH Image to ImageJ: 25 years of image analysis. Nat. Methods 9, 671–675 10.1038/nmeth.2089 22930834 PMC5554542

[B35] Steinhorst L. Kudla J. (2013). Calcium and reactive oxygen species rule the waves of signaling. Plant Physiol. 163, 471–485 10.1104/pp.113.222950 23898042 PMC3793029

[B36] Taylor K. L. Brackenridge A. E. Vivier M. A. Oberholster A. (2006). High-performance liquid chromatography profiling of the major carotenoids in *Arabidopsis thaliana* leaf tissue. J. Chromatogr. A 1121, 83–91 10.1016/j.chroma.2006.04.033 16701678

[B37] Verrillo F. Occhipinti A. Kanchiswamy C. N. Maffei M. E. (2014). Quantitative analysis of herbivore-induced cytosolic calcium by using a Cameleon (YC 3.6) calcium sensor in *Arabidopsis thaliana*. J. Plant Physiol. 171, 136–139 10.1016/j.jplph.2013.09.020 24331428

[B38] Ward W. W. Cormier M. J. (1976). *In vitro* energy transfer in *Renilla* bioluminescence. J. Phys. Chem. 80, 2289–2291 10.1021/j100561a030

[B39] Webb S. E. Rogers K. L. Karplus E. Miller A. L. (2010). The use of aequorins to record and visualize Ca^2+^ dynamics: from subcellular microdomains to whole organisms. Methods Cell Biol. 99, 263–300 10.1016/B978-0-12-374841-6.00010-4 21035690

[B40] Zhu X. Feng Y. Liang G. Liu N. Zhu J. K. (2013). Aequorin-based luminescence imaging reveals stimulus- and tissue-specific Ca2+ dynamics in Arabidopsis plants. Mol. Plant. 6, 444–455 10.1093/mp/sst013 23371933 PMC3603005

